# Consent in flux: when brain‑computer interface embodiment undermines informed consent

**DOI:** 10.1186/s12910-026-01540-1

**Published:** 2026-06-23

**Authors:** Yue Zhao, Yuan Lin

**Affiliations:** School of Law, Hainan Normal University, 99 Longkun South Road, Qiongshan District, Haikou, 571158 China

**Keywords:** Brain-computer interface, Informed consent, Extended cognition, Embodiment, Neurorights, Research ethics

## Abstract

**Background:**

In March 2026, China approved the first invasive brain‑computer interface (BCI) for clinical use globally. While this milestone accelerates neurotechnology translation, it exposes a neglected ethical problem: in some long-term invasive BCI cases, participants who initially consent to later device explantation may subsequently develop functional dependence on, and phenomenological incorporation of, the device. This paper examines whether such post-implantation integration can weaken the continuing moral authority of initial consent to explantation.

**Methods:**

This paper provides a normative analysis grounded in established bioethics concepts. We draw on theories of extended cognition and transformative experience, integrate findings from qualitative empirical studies of BCI user phenomenology, and engage with the neurorights discourse and Chinese regulatory developments. The analysis focuses on the moral authority of initial consent where long-term device integration may alter the participant’s embodied agency, with particular attention to the implications for interpreting the right to withdraw from research.

**Results:**

Transformative experience alone is not sufficient to distinguish implantable BCIs from other medical interventions that may also alter self-understanding. The distinctive diachronic consent problem arises when ex ante experiential ignorance is combined with extended cognition, embodied agency, functional dependence, and the possibility that explantation would remove a device experienced as part of the user’s agential system. We identify a specific governance gap in China’s current ethical guidelines and propose three interlocking reforms: adopting a dynamic consent model with periodic re‑consent encounters, establishing ongoing ethical review for implanted participants, and decoupling withdrawal from research from consent to explantation. An ethical algorithm to guide explantation decisions is presented.

**Conclusions:**

Informed consent for long-term invasive BCI use should be reconceptualised as an ongoing process where there is evidence of functional dependence or phenomenological incorporation. Initial consent remains important, but it should not be treated as conclusively authorising later explantation without post-integration reassessment.

## Background

### Brain‑computer interfaces: from research to routine care

Brain‑computer interfaces (BCIs) are neurotechnological devices that detect and measure brain activity and convert these neural signals into computer‑generated output, enabling users to perform tasks ranging from moving a cursor and operating computer programs to steering wheelchairs, controlling prostheses, or activating muscles [1–3]. Three types of BCIs can be distinguished according to the brain activity production employed: active BCIs require the user to perform a mental task such as motor imagery; reactive BCIs operate via selective attention to external stimuli; and passive BCIs measure ongoing brain activity without requiring voluntary performance [4]. The brain signals can be measured invasively, through electrodes implanted on the surface of the cortex or within cortical tissue, or non‑invasively, most commonly via electroencephalography (EEG) placed on the scalp [5, 6]. In virtually all BCI types, users receive real‑time feedback on their brain activity output through visual, auditory, tactile, vestibular, or proprioceptive channels [5].

The clinical promise of BCI technology is substantial. BCIs have been proposed as assistive technology for individuals who are non‑communicative or paralyzed, such as those with amyotrophic lateral sclerosis (ALS) or spinal cord injury [7, 8]. The technology enables locked‑in patients to communicate via BCI spellers [9, 10], allows individuals with paralysis to control prosthetic limbs and cursors [11, 12], and shows promise in neuro‑rehabilitation following stroke or spinal cord injury [13, 14]. Research has demonstrated additional applications including treatment of psychiatric conditions, epilepsy management, and cognitive enhancement [15, 16]. Beyond the medical domain, BCIs have also been suggested for entertainment and enhancement uses, with companies currently marketing BCI devices for gaming and personal cognitive monitoring [17].

In March 2026, a watershed moment arrived: China approved the first invasive brain‑computer interface for clinical use worldwide, namely the NEO hand motor compensation system developed by a team led by Professor Hong Bo at Tsinghua University [18, 19]. The approval followed 32‑36 successful clinical trials involving individuals with tetraplegia who demonstrated the ability to control robotic limbs via the implanted device [20]. The NEO system, which employs a minimally invasive epidural implantation technique with wireless power and data transmission, represents the first invasive BCI medical device to enter routine clinical application globally [21, 22]. BCI is now designated a strategic industry under China’s 15th Five‑Year Plan and 2026 Government Work Report, supported by coordinated regulatory, reimbursement, and clinical frameworks. This policy momentum signals a broader transition: BCI is moving from the domain of experimental research to that of commercially available medical devices, a transition that fundamentally alters the ethical landscape within which the technology operates [23].

### The ethical fault line beneath policy acceleration

The rapid policy development in China has been accompanied by notable advances in ethical governance. The 2024 Ethical Guidelines for Brain‑Computer Interface Research, issued by China’s National Science and Technology Ethics Committee, articulates principles of scientific soundness, beneficence, autonomy, minimum harm, privacy, and fairness and justice [24]. In July 2025, the Ministry of Science and Technology issued the Ethical Guidelines for Neurotechnology Medical Research Involving Human Subjects, which specifies ethical requirements for neural data collection, analysis, and neuromodulation research activities, including privacy protection, risk‑benefit assessment, and multiple risk prevention [25, 26]. Furthermore, in January 2026, China released the Multidisciplinary Ethics Review Guidelines for BCI Clinical Research, establishing review standards and elements tailored to the unique characteristics of invasive and non‑invasive BCI technologies [27]. At the international level, UNESCO adopted the world’s first Recommendation on the Ethics of Neurotechnology in November 2025, articulating principles to safeguard mental integrity and cognitive liberty [28].

Yet beneath this policy velocity lies an ethical fault line that has received almost no systematic attention in regulatory frameworks worldwide. Available cases and qualitative reports suggest that some clinical trial participants may develop a form of embodied integration with their implants after signing consent forms stating that “the device will be explanted at study conclusion.” When a device transitions from being an external tool to being experienced as part of one’s bodily and cognitive self, a transformation captured by the user who no longer says “I move the robotic arm” but “I moved my arm” [29], the moral authority of a pre‑implantation agreement to its removal becomes contestable. This is not a hypothetical concern: the case of Patient R, who described the non‑voluntary explantation of her seizure‑prediction BCI as losing “a part of me. that could never be replaced,” provides a stark precedent [30, 31]. Gilbert and colleagues have further documented how implanted BCIs can produce harms related to personality, identity, autonomy, authenticity, agency, and self (PIAAS), underscoring the depth of identity transformation at stake [32].

### The diachronic consent problem: an overview

This paper argues that invasive BCI research and clinical translation confront informed consent with what we term a diachronic consent problem: the “I” who consents ex ante is not identical, in phenomenologically and ethically relevant respects, to the “I” asked to surrender the device ex post. More precisely, a diachronic consent problem arises when the following conditions are jointly satisfied: (a) an agent provides ex ante consent to a future intervention (here, device explantation) under conditions of necessary experiential ignorance; (b) the intervention itself (BCI implantation) induces a transformation in the agent’s embodied self, such that the agent’s post‑intervention preferences, values, or identity diverge in normatively relevant respects from her pre‑intervention state; and (c) enforcing the original consent would cause the post‑intervention agent a harm that she could not have fully appreciated ex ante.

The argument advanced here is not that every transformative medical experience invalidates prior consent, nor that all BCI use necessarily produces a diachronic consent problem. Many medical interventions, including serious illness, mastectomy, organ transplantation, reproductive decisions, and psychiatric treatment, may be personally or epistemically transformative. What makes the case of long-term invasive BCI distinctive is the possible convergence of four features: direct neural coupling, durable functional dependence, phenomenological incorporation into embodied agency, and the possibility that device removal severs a component of the user’s extended agential system. The diachronic consent problem therefore arises most clearly in cases where there is evidence that the device has moved beyond being a tool and has become integrated into the user’s practical agency or self-experience.

Standard single-point consent frameworks, while capable of recognising changed preferences through the right to withdraw, provide limited guidance for determining whether prior consent to explantation remains authoritative after device integration has occurred. Drawing on theories of extended cognition, empirical studies of BCI user experience, and the emerging neurorights discourse, we contend that informed consent in the context of implantable BCIs must be reconceptualised as an ongoing process that adapts to the participant’s evolving embodied experience.

The paper proceeds as follows. First, we articulate the diachronic consent problem in detail, distinguishing two temporally distinct questions often conflated in ethical discourse: the initial validity of consent (Q1) and the ongoing validity of that consent after device integration (Q2). Second, we ground this problem in the phenomenology of BCI embodiment, drawing on extended cognition theory and qualitative empirical research to explain why device integration matters for the moral status of consent. Third, we argue that initial consent does not exhaust moral authority when the intervention transforms the agent, engaging with philosophical work on consent, transformative experience, and the right to withdraw. Fourth, we analyze the specific governance gap in China’s current ethical framework and situate this gap within the broader international literature on post‑trial obligations in neural device research. Fifth, we propose three interlocking reforms: adopting a dynamic consent model, establishing ongoing ethical review for implanted participants, and decoupling “withdrawal from research” from “consent to explantation.” Sixth, we present an ethical algorithm to guide explantation decisions in clinical and research contexts. We conclude by reflecting on the urgent need to build an ethical framework that honours the evolving embodied experience of BCI users, an imperative that grows more pressing as China and other nations accelerate toward commercial BCI deployment.

In their scoping review of BCI ethics literature, Burwell and colleagues identified that while ethical issues such as personhood, autonomy, and research ethics had been enumerated extensively, “few concrete recommendations have been expressed” [17]. The present study addresses this gap by providing a specific, actionable framework—comprising dynamic consent protocols, ongoing ethical review mechanisms, and a structured decision‑making algorithm—for managing the diachronic consent problem. By bridging previously disconnected literatures on BCI phenomenology [29], theoretical resources of extended cognition and transformative experience [33, 34], and expert and user expectations [35, 36], the analysis moves from problem identification to prescriptive reform.

## Methods

This study employs a normative analysis grounded in established bioethics concepts and frameworks. The methodology integrates three complementary approaches. First, we draw on philosophical theories, specifically the extended mind thesis [33] and the concept of transformative experience [34], to provide a theoretical foundation for understanding how BCI integration alters the agent’s embodied self. Second, we engage with qualitative empirical research on BCI user phenomenology [29, 35, 36] to ground the normative claims in documented first‑person experiences. Third, we conduct a critical examination of existing regulatory instruments, including China’s 2024 Ethical Guidelines for Brain‑Computer Interface Research [24] and the 2025 Neurotechnology Research Ethics Guidelines [25], to identify specific governance gaps. The normative analysis is structured by a hybrid ethical framework grounded in principlism, supplemented by consequentialist and deontological reasoning where appropriate. Principlism, as articulated by Beauchamp and Childress [37], provides the primary normative architecture through its four core principles: respect for autonomy, beneficence, non‑maleficence, and justice. The diachronic consent problem engages all four principles. Respect for autonomy is implicated because the participant’s contemporaneous preferences regarding explantation may conflict with her prior consent; beneficence and non‑maleficence are engaged in weighing the functional benefits of device retention against the medical risks of long‑term implantation; and justice concerns arise in the equitable allocation of post‑trial support resources. Where principlism alone yields indeterminate guidance, the analysis draws on supplementary normative resources: the extended mind thesis and transformative experience theory provide the descriptive foundation for understanding how BCI integration alters the agent’s embodied self; the neurorights framework offers a rights‑based language for articulating the stakes of non‑voluntary explantation; and consequentialist balancing informs the risk‑benefit assessment embedded in the ethical algorithm. This multi‑pronged approach is appropriate for a novel ethical challenge that resists resolution within any single normative framework.

More specifically, the analysis proceeded in four steps. The analysis first distinguishes initial consent to implantation from later consent to explantation, and then evaluates whether the concept of transformative experience can adequately explain this distinction. Because transformative experience alone cannot distinguish BCI explantation from other ethically significant medical experiences, the paper further draws on extended cognition and embodiment theory to specify what is distinctive about long-term invasive BCI use. On this basis, it develops governance implications for dynamic consent, continuing ethical review, and explantation decision-making. The focus is therefore on the normative structure of consent under conditions of technological embodiment, rather than on estimating the empirical prevalence of BCI-related identity transformation.

The qualitative empirical studies on BCI user phenomenology cited in this analysis were identified through a purposive sampling strategy targeting peer‑reviewed publications that directly address the first‑person experience of BCI use. Priority was given to studies employing semi‑structured interviews or focus group methodologies that captured the lived experience of BCI users with physical impairments, as this population is most directly affected by the diachronic consent problem. The regulatory instruments analyzed include three Chinese BCI ethics guidelines issued between 2024 and 2026, selected because they constitute the most comprehensive and recent articulation of China’s BCI governance framework. The analysis of these instruments employed qualitative content analysis focusing on the presence or absence of provisions addressing post‑trial device management and ongoing consent procedures.

## Results

### The diachronic consent problem: two temporally distinct questions

#### The presumption of agent stability in standard consent frameworks

Informed consent doctrine, as codified in international research ethics guidelines including the Declaration of Helsinki and the Council for International Organizations of Medical Sciences (CIOMS) guidelines, treats consent as a one‑time authorisation that remains valid unless explicitly withdrawn [38, 39]. Standard consent frameworks do not deny that preferences may change over time. The right to withdraw from research explicitly recognises that participants may later revise their decisions. However, these frameworks usually treat such revision as a change in preference rather than as a change in the normative relationship between the participant and the intervention itself. They provide limited guidance for cases in which the intervention becomes incorporated into the participant’s embodied agency and where later removal may no longer be equivalent to simply ending participation in a study. The problem is therefore not that standard consent frameworks assume absolute psychological stability, but that they lack a structured mechanism for assessing whether technologically mediated embodiment alters the continuing authority of prior consent to device removal.

Long-term invasive BCIs, however, may differ from many medical interventions in their capacity to become functionally and phenomenologically incorporated into the user’s embodied agency. Unlike interventions that primarily alter bodily condition or future life prospects, an invasive BCI can directly interface with neural activity and, through sustained use, become a reliable means through which the user communicates, acts, or interacts with the environment. Where such incorporation occurs, later device removal may no longer be equivalent to discontinuing an external tool or ending participation in a study. It may instead affect a component of the user’s practical agency and self-experience. As Klein and colleagues have observed, neural devices can “engineer the brain” in ways that raise distinctive ethical issues not adequately captured by frameworks developed for other medical technologies [40]. The technology’s unique capacity to alter the agent’s embodied self generates a problem that standard consent frameworks are ill‑equipped to address. A striking peculiarity of BCI technology, as Steinert and colleagues note, is that the kind of actions it enables seems to differ from paradigmatic human actions because effects in the world are brought about by devices directed by brain signals, and the sequence need not involve bodily or muscle movements at all [41]. This disembodied nature of BCI‑mediated events carries important implications for how we understand agency in this context and, consequently, for the moral standing of consent.

#### Q1 and Q2: distinguishing two normative questions

To bring analytical clarity to this problem, we distinguish two distinct ethical questions that are often conflated in discussions of BCI trial consent:

##### Q1. The initial validity question

At the time of signing, did the participant provide sufficiently informed and voluntary consent to implantation and eventual explantation? This question concerns the adequacy of pre‑implantation disclosure, the participant’s capacity to understand the disclosed information, and the voluntariness of the decision to participate. It is the question that has dominated regulatory attention and research ethics discourse to date.

##### Q2. The ongoing validity question

As the device becomes integrated into the participant’s cognitive and bodily self, does the initial consent to explantation retain its moral authority? This question concerns the normative force of a consent given from a position of necessary experiential ignorance after the participant has acquired the relevant experiential knowledge, namely knowledge of what it is like to live with the device as part of one’s embodied self.

This distinction does not imply that pre-implantation consent is invalid. A participant may validly consent to implantation, research participation, surgical risks, data collection, and even the protocol’s anticipated endpoint on the basis of the information available at Q1. The claim is narrower: Q1 consent may be insufficient to conclusively determine Q2 where the participant later acquires morally relevant experiential knowledge through sustained device use. In this sense, the diachronic consent problem is not a wholesale objection to initial informed consent, but a challenge to treating initial consent as a final authorisation for later explantation after functional and phenomenological integration has occurred.

The distinction between Q1 and Q2 is not merely analytical; it tracks a genuine normative difference. One can imagine a scenario in which Q1 is answered affirmatively, meaning the pre‑implantation consent was impeccably informed and voluntary, yet Q2 is answered negatively, meaning the post‑implantation agent has undergone such a significant transformation that the original consent to explantation no longer binds. Indeed, as we will argue, the very success of BCI in restoring agency and function creates the conditions under which initial consent to explantation may lose its moral footing.

#### Transformative experience, extended cognition, and the specific case of BCI explantation

Philosophical accounts of consent recognise consent as an ongoing normative state that requires continuing endorsement. As Archard argues, consent remains valid only if the agent continues to endorse it under unchanged conditions [42]. The key question, then, is whether BCI implantation constitutes a relevant change in conditions, a change that alters the normative status of the original consent. We contend that it does, precisely because it alters the agent’s embodied self.

Paul’s account of transformative experience is useful, but only in a limited role. It explains why a participant may lack ex ante experiential knowledge of what it will be like to live with, depend on, and potentially lose an implanted BCI. However, transformative experience alone cannot distinguish BCI explantation from many other medical or life experiences that may also reshape preferences and self-understanding. Serious illness, disability, mastectomy, childbirth, and psychiatric treatment may all be transformative in relevant respects. The distinctiveness of long-term invasive BCI does not lie merely in transformation, but in the possible incorporation of the device into the user’s embodied and agential system.

The argument therefore relies on the combination of transformative experience and extended cognition. Transformative experience identifies the epistemic limitation of initial consent; extended cognition and embodiment explain why the later object of decision may have changed. At Q1, the participant consents to the future removal of a device understood as an implanted medical technology. At Q2, the participant may confront the removal of something that has become functionally relied upon, phenomenologically transparent, and experienced as part of the user’s agency. This is what distinguishes the BCI case from many other transformative medical experiences.

This combined account also clarifies the status of pre-implantation consent. The claim is not that experiential ignorance makes Q1 consent invalid. Rather, Q1 consent may be valid for implantation, research participation, surgical risks, data collection, and the protocol’s anticipated endpoint, while still being insufficient to conclusively settle Q2. The ethically significant issue is whether sustained device use has generated forms of dependence, incorporation, or self-experience that were unavailable to the participant at the time of initial consent and that bear directly on the permissibility of later explantation.

#### Empirical corroboration of the epistemological gap

Expert interviews in China also suggest that even technically informed observers may not fully anticipate the first-person experience of long-term BCI use, reinforcing the need to treat post-implantation experience as ethically relevant rather than merely anecdotal [35]. Crucially, even technical experts, individuals with deep knowledge of BCI mechanisms and limitations, struggle to anticipate the subjective, experiential dimension of long‑term device integration. Their expertise provides no privileged access to the phenomenological “what it is like” of BCI use.

Kögel and colleagues’ scoping review found that social research on BCIs has focused more on usability, satisfaction, and quality of life than on the user’s first-person experience of BCI as it relates to identity, agency, and self-experience [43]. This gap matters because Q2 depends precisely on whether the device has become incorporated into the participant’s practical agency or self-relation [43].

This lack of empirical attention to the first‑person experience of BCI use compounds the epistemological asymmetry at the heart of the diachronic consent problem. We are asking participants to consent to device removal without having systematically investigated what it feels like to live with, and then lose, an integrated BCI. The ethical imperative to address Q2 is thus not merely theoretical; it is grounded in an empirical gap that limits our understanding of what is at stake for BCI users.

#### The gap in Chinese regulatory attention

Most regulatory attention globally has focused on Q1. China’s 2024 Ethical Guidelines for Brain‑Computer Interface Research emphasises pre‑trial disclosure and the right to withdraw at any time [24]. The guidelines specify that informed consent must respect “disclosure, capacity, and voluntariness,” and that participants must be fully informed of the purpose, methods, potential risks, and benefits of the research. The 2025 Neurotechnology Research Ethics Guidelines further elaborate requirements for risk‑benefit assessment and privacy protection [25]. The 2026 Multidisciplinary Ethics Review Guidelines establish standards for evaluating BCI clinical research protocols [27].

But Q2 remains unaddressed across all these instruments. None of the guidelines specify how withdrawal should be operationalised when the intervention is an implanted device whose removal is itself a significant medical procedure with psychological sequelae. None address the possibility that a participant’s preferences regarding explantation may evolve over time as the device becomes integrated into her embodied self. None provide a framework for evaluating whether the initial consent to explantation retains its moral authority after months or years of device integration. This is a critical oversight, because the very success of BCI in restoring agency and function creates the conditions under which initial consent to explantation may lose its moral footing.

### Why BCI embodiment matters: the extended self

#### Extended cognition and the incorporation of technology

Why take the diachronic consent problem seriously? The answer lies in the distinctive way invasive BCIs can become incorporated into the user’s body schema and cognitive architecture, a process that philosophers and cognitive scientists have described under the rubric of “extended embodiment” and “extended cognition.”

The theoretical relevance of extended cognition lies in identifying when a device ceases to function merely as an external tool and becomes integrated into the user’s action system. In the BCI context, this possibility is supported by evidence of reliable neural coupling, functional dependence, transparency in use, and first-person reports of direct agency.

In the context of BCIs, Aas and Wasserman distinguish between two ways of construing BCI‑connected devices: as external tools or as parts of an extended body [44]. When a BCI functions as a mere tool, operated with conscious effort and external to one’s sense of self, its removal carries no more existential weight than replacing a malfunctioning keyboard. The user thinks “I am using this device to accomplish a task,” and the device remains phenomenologically distinct from the self. But when a device achieves high levels of functional integration, direct voluntary control, and sensory feedback, it may cross a phenomenological threshold and become “incorporated.” At this point, the device is no longer experienced as something I use but as something I am. The authors note that this distinction is not merely conceptual but carries significant implications for how we understand the user’s relationship to the technology and the normative significance of device removal.

Cassinadri and Ienca apply extended mind theory to the case of Patient R, whose implanted BCI predicted epileptic seizures and alerted her to take medication [30]. They argue that her BCI satisfied the criteria for cognitive extension, including permanent coupling, informational integration, trust, and functional dependence, making it a constitutive component of her extended cognitive system. The device was not merely a tool she used; it had become, in a functionally and phenomenologically robust sense, part of her cognitive architecture. This analysis provides a theoretical framework for understanding why non‑voluntary explantation can constitute a harm that goes beyond mere inconvenience or loss of function: it severs the agent from a component of her extended self.

#### Phenomenological evidence from BCI users

This is not mere philosophical speculation. Qualitative empirical research with BCI users provides compelling phenomenological evidence of device incorporation. Kögel and colleagues’ interview study with nine BCI users found that participants consistently described successful BCI operation in terms of direct agency [29]. One participant, Nicole, described the transformation: “I think it was the second day of training when [the robotic arm] became my arm. They stopped saying ‘I move the robotic arm.’ Then I started saying, ‘I moved my arm.’ Not ‘Look what the arm did,’ but ‘Look at what I did.’ Instead of saying, ‘I moved the computer right,’ I would say, ‘I moved right’ because it became my arm. I felt like it was part of me.”

Notably, when the BCI produced errors, users in Kögel and colleagues’ study tended to attribute responsibility to themselves rather than the machine, a hallmark of embodied agency in which the technology becomes transparent in skilled use. One participant explained: “When there was a wrong command, I got cross with myself, because I thought somehow ‘Oh, apparently I didn’t concentrate properly.’” This attribution pattern, blaming oneself for the technology’s failures, mirrors how we relate to our biological bodies when they fail us. The technology has become, in the user’s phenomenological experience, part of the agential system for which she takes responsibility. As Vlek and colleagues have observed, performing an action with the assistance of a BCI may affect a user’s judgment of agency, potentially resulting in an illusion of control [45]. van de Laar and colleagues’ experimental work further demonstrates that the relationship between actual and perceived control in BCI systems is complex and non‑linear, with implications for how users experience agency [46].

The study also revealed a striking phenomenological dimension of BCI use: the imperative of emotional suppression. One participant described: “You are NOT allowed to have emotions, none whatsoever. You completely have to be dead inside.” This account reveals both the depth of cognitive engagement required for successful BCI operation and the extent to which the device becomes integrated into the user’s agential experience. The user must modulate her own affective states to maintain device performance, a form of self‑regulation that blurs the boundary between the user’s mental life and the technology’s functional requirements.

Schicktanz and colleagues’ qualitative research with potential BCI users confirms the epistemological gap [36]. Individuals considering BCI implantation tend to focus on functional gains, including independence, communication, and privacy, and rarely anticipate the depth of identity integration that may ensue. Participants in their study hoped for more independence and self‑control, while some expressed concerns about data protection or feared creating “self‑transcending human‑machine hybrids,” but none articulated an expectation that the device might become phenomenologically incorporated into their sense of self. This finding underscores the structural nature of the epistemological limitation: even when participants are thoughtfully engaged in considering the implications of BCI use, they cannot access the experiential knowledge that would enable them to fully grasp what is at stake in eventual device removal.

#### Implications for the moral authority of initial consent

These phenomenological findings do not show that initial consent is invalid. They show why initial consent should not be treated as the final word on explantation once device integration has occurred. Where the participant later experiences the device as part of her agency, the relevant ethical question becomes whether the earlier consent still carries sufficient authority to justify removal.

This epistemological asymmetry is not a failure of the consent process that could be remedied by better disclosure. It is a structural feature of the transformative experience of BCI use. As Klein has argued in her analysis of informed consent in implantable BCI research, “the emerging and rapidly evolving nature of implantable BCI research complicates the identification of risks, a critical component of informed consent” [47]. The risks associated with device removal, including the loss of a component of the extended self and the attendant psychological distress, cannot be fully appreciated ex ante. They can only be understood ex post, after the participant has experienced life with an integrated BCI.

### Why initial consent does not exhaust moral authority

#### The provisional nature of pre‑implantation consent

If the preceding analysis is correct, the moral authority of pre‑implantation consent to device removal does not persist unchanged throughout the implantation period. Initial consent must be understood as provisional with respect to explantation, subject to reaffirmation or revision once the participant has acquired relevant experiential knowledge.

This understanding aligns with broader critiques of single‑point consent in biomedical research. Wexler and Feinsinger argue that communication about BCI risks and benefits must be ongoing, as patients’ expectations evolve with experience [48]. They note that “consent is not a one‑time event but a process that should continue throughout the research study and beyond,” and they call for “ongoing communication and re‑consent” as participants’ understanding deepens. Klein similarly advocates a “reconsent” process, noting that BCI data collection differs from traditional treatments and that participants’ understanding of what is at stake evolves over time [47].

Wang Wenyu, analysing the dynamic expansion of BCI applications in China from therapeutic to enhancement purposes and from non‑invasive to invasive modalities, specifically identifies the “hollowing out” of informed consent as a key risk [49]. He argues that the shift from non‑invasive to invasive BCIs means that “the user’s ability to know the risks and to withdraw from them at any time will be weakened, leading to the hollowing out of the right to informed consent.” While Wang focuses primarily on the difficulty of risk disclosure and the practical obstacles to device removal, his analysis underscores the structural vulnerability of consent in the invasive BCI context and points toward the need for more robust, ongoing consent mechanisms.

The right to withdraw: conceptualising withdrawal from neural device research.

The diachronic consent problem has direct implications for interpreting the right to withdraw from research. Paragraph 26 of the Declaration of Helsinki states that “the potential subject must be informed of the right to refuse to participate in the study or to withdraw consent to participate at any time without reprisal” [38]. But what does “withdraw consent” mean when the intervention is an implanted device whose removal is itself a significant medical procedure with psychological sequelae?

The standard interpretation, which holds that withdrawal entails explantation, presupposes that the participant’s interest lies in returning to a pre‑implantation state. But if the device has become part of the extended self, the participant may have a profound interest in retaining the device even while withdrawing from the research protocol. Conflating “withdrawal from research” with “consent to explantation” thus denies participants a meaningful choice at the very moment when their experiential knowledge would enable them to make an authentically informed decision. The right to withdraw, as traditionally conceptualized, assumes a binary choice: either remain in the study or return to the pre‑study condition. For implantable BCIs, however, a third possibility emerges—withdrawal from the research protocol while retaining the implanted device with appropriate ongoing support—which the standard binary model fails to accommodate.

Hendriks and colleagues, in their comprehensive review of ethical challenges in human research with neural devices, identify informed consent and post‑trial responsibilities as central concerns [50]. They note that “neural devices research involves unique risks and uncertainties that challenge traditional conceptions of informed consent,” and they call for “ongoing consent processes that acknowledge the evolving nature of participants’ experiences and the potential for changing preferences over time.” This analysis reinforces the need to reconceptualise consent in neural device research as a dynamic, ongoing process rather than a single authorisation.

#### Objection: could advance directives bind the future self?

One might object that advance directives could bind the future self. If the participant agreed ex ante to explantation, why privilege her later change of heart? The relevant point is not that the post-implantation participant is necessarily more autonomous than her pre-implantation self. Rather, she should not be presumed to have diminished autonomy merely because her preferences have changed after device integration. If she retains decision-making capacity, her contemporaneous preference ordinarily deserves substantial weight. Advance directives in medical contexts are generally not treated as binding against a presently competent person who has changed her mind. By analogy, an initial statement accepting later explantation should not automatically override a competent post-implantation participant’s later, experience-informed preference.

Furthermore, as Hurst and Bobier argue in their critique of Ulysses contracts in BCI research, such contracts should not be used to override the experience-informed preferences of competent participants who later change their minds about explantation [51]. The diachronic consent problem we identify is precisely an instance of this broader concern: the transformation wrought by BCI integration calls into question the authority of the past self to bind the present self.

#### Neurorights as a normative framework

The neurorights framework offers a normative language for articulating what is at stake in the diachronic consent problem. Ienca and Andorno have proposed four core neurorights: cognitive liberty, mental privacy, mental integrity, and psychological continuity [52]. These rights are grounded in established human rights frameworks but are specified to address the distinctive challenges posed by neurotechnology. Cognitive liberty protects the right to make free and competent decisions regarding one’s own neural activity; mental privacy protects against non‑consensual access to neural data; mental integrity protects against non‑consensual alterations to mental states; and psychological continuity protects against disruptions to one’s sense of self over time.

Cassinadri and Ienca argue that non‑voluntary BCI explantation violates multiple dimensions of these rights, particularly the right to mental integrity and the right to psychological continuity, because it severs the participant from a constitutive component of her extended cognitive system [30]. The loss is not merely functional; it is existential. The participant loses not only a tool but a part of herself.

It is important to acknowledge that the neurorights framework remains contested. Bublitz, in a careful analysis, argues that while neurorights proposals articulate genuine normative concerns, many of these concerns can be addressed within existing rights frameworks, such as privacy, bodily integrity, and freedom of thought, without creating novel rights categories [53]. He contends that “novel neurorights are not conceptually necessary” because existing rights “already protect against the core threats” posed by neurotechnology. This debate about the novelty of neurorights is important, but it does not undermine the core normative claim that matters for our argument: whatever rights framework is adopted, it must be interpreted dynamically in light of the transformative nature of BCI integration.

Li’s proposal can be read as a Chinese doctrinal translation of concerns that the international neurorights literature frames in terms of mental integrity and psychological continuity. Li argues that existing protections of bodily integrity and privacy may not fully capture the threats that BCI technologies pose to mental integrity [54]. Whereas Ienca and Andorno articulate these interests as emerging human rights claims, Li asks how similar interests might be accommodated within China’s existing personality-rights and civil-law framework. This connection matters for the present argument because the diachronic consent problem does not depend on whether a jurisdiction recognises “neurorights” as a separate legal category. The relevant ethical concern may be expressed through different legal vocabularies: mental integrity and psychological continuity in international neuroethics, personality interests and life dignity in Chinese civil law, or bodily integrity and decisional autonomy in other legal systems. What these frameworks share is the need to take seriously the possibility that non-voluntary explantation may affect more than bodily tissue; it may disturb the user’s self-relation and continuity of agency.

### The Chinese governance gap in international perspective

#### China’s BCI policy landscape

China’s rapid BCI policy advancement has created a regulatory framework that addresses Q1 with increasing sophistication but remains silent on Q2. This gap is particularly concerning given the number of Chinese patients who have already received investigational BCI implants and the possibility of broader clinical or commercial deployment following the NEO system’s approval.

The policy infrastructure for BCI in China has developed with remarkable speed. The 2024 Ethical Guidelines for Brain‑Computer Interface Research establish principles of scientific soundness, beneficence, autonomy, minimum harm, privacy, and fairness and justice [24]. The 2025 Neurotechnology Research Ethics Guidelines provide detailed requirements for neural data protection and risk‑benefit assessment [25, 26]. The 2026 Multidisciplinary Ethics Review Guidelines for BCI Clinical Research establish review standards tailored to BCI technologies [27]. In August 2025, seven government departments jointly issued Implementation Opinions on Promoting the Innovative Development of the Brain‑Computer Interface Industry, which call for “strengthening security safeguards, continuously promoting ethical research, establishing a data governance framework, regulating the collection, storage, and use of user information, preventing brain privacy leakage, and enhancing biosafety and digital information security protection capabilities” [55].

In the Chinese legal context, these general themes can be mapped onto three more concrete regulatory questions. First, autonomy concerns correspond to the rules on informed consent in biomedical research ethics and the Civil Code’s protection of bodily integrity and personality interests. Second, post-trial support concerns correspond to the responsibilities of research institutions, hospitals, sponsors, and device manufacturers under clinical research governance and medical device regulation. Third, psychological continuity and mental integrity concerns reveal a gap between existing protections of bodily integrity and privacy, on the one hand, and the less developed protection of identity-related and agency-related harms, on the other. The Chinese case is therefore not merely an example of global BCI ethics; it shows how global neuroethical concerns must be translated into institutionally specific duties and review procedures.

Yet none of these instruments address the question of what happens when a participant who has developed embodied integration with a BCI no longer wishes to have it removed, or when the participant’s preferences regarding explantation evolve over time. The 2024 Guidelines require that researchers “fully inform subjects of the purpose, methods, potential risks, and benefits of the research” and respect the right to withdraw “at any time.” But they do not specify how withdrawal should be operationalised when the intervention is an implanted device, nor do they address the possibility that a participant’s preferences regarding explantation may evolve. The 2025 Neurotechnology Guidelines focus primarily on data protection and risk assessment, with limited attention to post‑trial device management. The 2026 Multidisciplinary Ethics Review Guidelines establish procedural standards for initial review but do not address ongoing review of implanted participants or provide guidance for explantation decisions.

#### The pricing signal and institutional presumption

This silence is mirrored in pricing. The listing of “invasive BCI explantation” as a reimbursable procedure in Hubei Province at 3139 RMB may, in the absence of a parallel retention pathway, contribute to an institutional default toward removal. Though administratively necessary for reimbursement purposes, this standardised fee, coupled with the absence of any corresponding framework for post‑trial device retention, creates an institutional presumption of removal. For participants who have developed embodied integration with their BCIs, this presumption may exert subtle pressure to “consent” to removal, a form of structural coercion that undermines the voluntariness of the decision.

Ji Dongmei has systematically analysed the risks BCI technology poses to personality interests within China’s legal framework [56]. She argues that neural data is highly sensitive and difficult to de‑identify, requiring clarification within traditional legal frameworks. Moreover, she contends that current informed consent rules, information processing standards, and regulatory frameworks fall short of addressing the privacy threats inherent in neurotechnology. Ji advocates for a “full lifecycle supervision” approach that extends from research and development through to post‑market surveillance, a framework that resonates strongly with our proposal for ongoing ethical review.

Zhang Hong, analysing BCI through the lens of China’s Civil Code, contends that the “life dignity” protected under Article 1002 can be interpreted to encompass the self‑cognitive crises induced by BCI explantation [57]. She argues that self‑cognitive crisis, the feeling of “not being a living person” reported by some explantees, should be recognised as an infringement of the right to life dignity, and that BCI providers have an obligation not only to ensure therapeutic efficacy but also to ensure that patients psychologically accept their transformed state. These doctrinal innovations, while promising, remain at the level of academic commentary and have not been translated into regulatory practice.

#### Post‑trial obligations in neural device research: international evidence

The international evidence discussed below should be understood as illustrative rather than globally representative. Much of the empirical stakeholder literature on post-trial obligations in implanted neural device research has been generated in North American and European research settings. These studies cannot be assumed to represent all cultural or regulatory contexts. Their relevance to the Chinese case lies in identifying recurring ethical problems—maintenance, abandonment, cost allocation, and participant preferences after implantation—that may arise wherever long-term implanted neural devices are used. The question of post‑trial obligations in neural device research has garnered increasing international attention, providing important context for understanding the Chinese governance gap. Lázaro‑Muñoz and colleagues conducted a seminal study examining the perspectives of 44 stakeholders, including 23 researchers and 21 patient‑participants, involved in implantable neural device trials [58]. They found that “there is no clear standard regarding whether patients should retain or explant investigational neural devices at the end of a trial,” and that the absence of established infrastructures for ongoing care creates a regulatory gap that leaves implanted participants vulnerable to what they term “abandonment.” They note that implanted BCIs require ongoing maintenance, including battery replacement, recalibration, and software updates, and that without clear plans for post‑trial support, participants face a difficult choice between retaining a device that may fail without support or undergoing explantation.

Vooijs and colleagues’ systematic review of ethical, legal, and sociocultural considerations in neural device explantation, published in 2025, analysed 63 publications and identified key themes including “patient autonomy, informed consent, and the right to device removal; post‑trial access and care; device maintenance and failure; and the psychological impact of explantation” [59]. They found that “current regulatory frameworks often lack explicit guidance on explantation procedures and post‑trial responsibilities,” and they called for “the development of standardised protocols for explantation decision‑making that incorporate patient perspectives and recognise the potential for device integration into identity.” This systematic review reinforces our core argument: the question of explantation is not merely a technical or administrative matter, but an ethical issue with profound implications for patient autonomy and well‑being.

Cabrera and Weber further argue that justice considerations, including access, equity, and post‑trial care, must be elevated in BCI ethical priority‑setting [23]. They note that research participants in studies involving implantable BCIs “may typically lose access to the technology at the end of a study,” and that the situation is particularly complicated for trial devices that must be explanted after completion. They call for “detailed plans for providing care for participants at and beyond the end of the study period.” This call resonates strongly with the Chinese context, where rapid commercialisation may outpace ethical infrastructure.

Goering and colleagues articulate a compelling argument for “moral entanglement” as a source of post‑trial obligations in neural device research [60]. They argue that when researchers witness how a device improves a participant’s quality of life and becomes integrated into their identity, they incur obligations that transcend the standard researcher‑participant relationship. Moral entanglement is not invoked here merely as a theme in BCI discourse. It helps explain why investigators, sponsors, and institutions may acquire responsibilities that are not exhausted by the original protocol. When a research team facilitates implantation, trains the participant to use the device, observes the participant’s functional gains, and knows that the device has become central to her daily agency, the relationship is no longer adequately captured by a simple researcher-participant transaction. This does not mean that researchers must always provide indefinite device support. It does mean that decisions about explantation, maintenance, and transition of care should be made through a process that recognises the participant’s dependence and the institution’s role in creating it.

Comparative analysis with international frameworks further illuminates the Chinese governance gap. The UNESCO Recommendation on the Ethics of Neurotechnology [28] explicitly calls for “mechanisms to ensure continuity of care and support for users of neurotechnology devices beyond the research period.” The European Union’s General Data Protection Regulation (GDPR) classifies neural data as a special category of personal data requiring heightened protection, though it does not specifically address device retention. The U.S. BRAIN Initiative’s Neuroethics Working Group has similarly emphasized the need for “clear post‑trial plans that address device maintenance, explantation procedures, and ongoing support.” While these international instruments provide valuable guidance, they remain largely aspirational and lack the concrete procedural mechanisms needed to operationalise post‑trial obligations in clinical and research settings.

## Discussion

The preceding analysis has established that standard single‑point consent frameworks cannot adequately address the diachronic consent problem posed by implantable BCIs. In this section, we elaborate on the implications of our findings, propose concrete reforms, and address potential objections to our approach.

### From single‑point to dynamic consent: a proposal

#### Three interlocking reforms

To address the diachronic consent problem in the context of China’s rapidly evolving BCI landscape, and with relevance for BCI research and clinical translation globally, we propose three interlocking reforms.

##### First, adopt a dynamic consent model

Dynamic consent treats consent not as a one‑time event but as an ongoing communicative process that adapts to changes in the participant’s circumstances and understanding [61]. In the BCI context, this would require periodic “re‑consent encounters” at predetermined milestones:

1. After the participant has achieved reliable device control (typically several weeks to months post‑implantation);
2. When the participant reports that the device has significantly impacted daily life or self‑experience;
3. As the trial’s scheduled endpoint approaches (or, in the clinical context, at regular intervals such as annually);
4. Whenever the participant expresses a desire to retain or remove the device.

These milestones are not arbitrary. They correspond to ethically salient transitions in the participant’s relationship with the device. Reliable device control marks the point at which the BCI may begin to shift from an experimental apparatus to a usable means of action. Reported effects on daily life or self-experience indicate that the device may be acquiring functional or phenomenological significance. The scheduled trial endpoint is the moment at which prior consent to explantation would otherwise be operationalised and therefore requires renewed scrutiny. Finally, any expressed desire to retain or remove the device should trigger reassessment because it provides direct evidence that the participant’s experience-informed preference may have changed. These points are intended as minimum triggers; they do not preclude additional reassessment where clinically or ethically indicated.

At each encounter, the participant’s current understanding, preferences, and degree of device integration would be assessed, and consent to continued participation and eventual explantation would be reaffirmed or revised accordingly. The assessment should include both structured questions about functional dependence and open‑ended exploration of the participant’s phenomenological experience of the device. Open-ended exploration of phenomenological experience is necessary not to satisfy researchers’ curiosity, but to identify ethically relevant facts that structured risk forms may miss. These include whether the participant experiences the device as transparent in use, whether she attributes device-mediated action to herself, whether removal is anticipated as a loss of agency rather than merely a loss of equipment, and whether the prospect of explantation produces identity-related distress. Such information directly bears on the ethical assessment of integration, voluntariness, and the proportionality of explantation. This approach recognises that the agent whose consent matters is the present agent, with her embodied experience, not the past agent who signed under necessary ignorance. Autonomy is not exercised once and for all; it is an ongoing capacity for self‑governance that must be supported throughout the research and clinical trajectory.

Dynamic consent has been promoted as a promising approach for biobanks and data‑intensive medical research, enabling participants to maintain control over the use of their biospecimens and health data over time [62]. While the BCI context differs from biobanking in important respects, given that the intervention is physical rather than informational, and the stakes involve embodied identity rather than data privacy, the underlying rationale for dynamic consent applies with equal force: the participant’s understanding, preferences, and circumstances evolve, and the consent process must evolve accordingly.

##### Second, establish ongoing ethical review for implanted participants

Current Chinese ethics review practice focuses on initial protocol approval and periodic continuing review, typically at annual intervals. For BCI trials and clinical implantation, we recommend that ethics committees establish a “tracking review” mechanism with shorter intervals and specific attention to device integration and participant preferences regarding explantation. The tracking review should include:

1. Independent assessment of the participant’s cognitive and emotional state;
2. Evaluation of the degree of functional dependence on the device, potentially using validated instruments such as the Psychosocial Impact of Assistive Devices Scale (PIADS) [63] or the Quebec User Evaluation of Satisfaction with assistive Technology (QUEST 2.0) [64] to quantify integration;
3. Documentation of any expressed concerns about device removal;
4. Assessment of the feasibility of continued device support if retention is contemplated.

In the Chinese context, this assessment need not require an entirely new institution in every case. It could be organised as an ad hoc BCI ethics consultation panel under the existing institutional ethics committee of the hospital or research institution. The panel should include, where available, a neurosurgeon or neurologist familiar with the device, a rehabilitation specialist, a psychologist or psychiatrist, a medical ethicist, a device engineer or technical representative, and a member independent of the research team. For high-risk or commercially sponsored trials, regional ethics review collaboration mechanisms or specialised neuroethics committees could provide external consultation. The panel’s task would not be to replace the treating team, but to provide an independent assessment of capacity, device integration, psychological vulnerability, and the feasibility of continued support. Its conclusions should be documented as part of continuing review, protocol modification, or post-trial care planning.

Where the participant’s preferences conflict with the trial protocol’s explantation plan (or, in the clinical context, where the participant resists clinically indicated removal), the committee should convene an ad hoc review to determine whether the plan should be modified in the participant’s best interests. This review should include input from the participant (or surrogate), the clinical team, an independent ethics consultant, and, where appropriate, a psychologist or psychiatrist familiar with the psychological dimensions of BCI use.

This approach aligns with Ji Dongmei’s “full lifecycle supervision” framework, which extends from research and development through to market withdrawal with adaptive feedback loops [56], and with Wang Wenyu’s “waterfall supervision” model of iterative stage review [49]. It also resonates with international calls for enhanced post‑trial oversight in neural device research. Lázaro‑Muñoz and colleagues specifically recommend that “funders and regulators should require that investigators have a clear plan for post‑trial access and device maintenance” [58]. Ongoing ethical review provides a mechanism for ensuring that such plans are not only articulated but implemented and adapted as circumstances evolve.

##### Third, decouple “withdrawal from research” from “consent to explantation”

Participants should be explicitly informed, at both initial consent and each re‑consent encounter, that withdrawal from the research protocol does not automatically entail device removal. The decision to retain or remove the device should be treated as a distinct clinical and ethical determination, made in consultation with the participant (or surrogate), the clinical team, and an independent ethics consultant.

A default presumption in favour of retention could be established for cases where the device has become integrated into the participant’s embodied self, rebuttable only by clear evidence that retention poses a significant and unmanageable health risk. This presumption would operationalise the insight that the participant’s interest in bodily and mental integrity, protected under both Chinese law and international human rights instruments, may weigh more heavily than the investigator’s interest in a clean study termination. It would also provide a structured pathway for addressing the “moral entanglement” that Goering and colleagues identify as a source of post‑trial obligations [60].

This decoupling would require revisions to standard consent form language. Rather than stating “the device will be removed at the end of the study,” consent forms should state: “The study protocol currently plans for device removal at [timeframe]. However, we recognise that your experience with the device may change how you feel about removal. You have the right to discuss alternatives to removal with the research team and ethics committee at any time, and the final decision regarding retention or removal will be made in consultation with you, taking into account your preferences, safety considerations, and the feasibility of ongoing support.”

### Risks of a default retention presumption

While the preceding argument establishes a presumption in favour of retention for integrated devices, this presumption is rebuttable and must be accompanied by robust safeguards. Several categories of risk warrant explicit acknowledgment. First, medical risks: long‑term implantation carries ongoing risks of infection, electrode migration, and tissue encapsulation, which may increase over time. The Step 2 safety assessment in the proposed algorithm is designed to identify cases where these risks outweigh the benefits of retention. Second, device obsolescence and maintenance: retained devices may become unsupported if the manufacturer ceases operations or the research team disbands. The Step 4 feasibility assessment requires a clear plan for ongoing technical support before retention is approved. Third, financial risks: post‑trial device maintenance incurs costs that must be allocated to a responsible party. The “clear designation of responsible parties” requirement in Step 4 addresses this, but regulatory clarity on cost‑sharing remains a broader policy need. Fourth, foreclosure of future options: retaining an older device may complicate the implantation of newer devices. Fifth, psychological dependency: the device may become so integrated that the participant’s sense of self becomes pathologically dependent on its continued function. The default presumption in favour of retention is not an absolute rule but a starting point for deliberation, rebuttable by any of the countervailing considerations enumerated here. The algorithm’s stepwise structure ensures that these risks are systematically evaluated before a retention determination is finalized.

### An ethical algorithm for explantation decisions

To guide ethics committees and investigators in navigating explantation decisions for participants who have developed embodied integration with their BCIs, we propose the following decision‑making framework, adapted from the algorithm developed by Pugh and colleagues for ethical analysis of complex research scenarios [65]:

- Step 1: Assess integration level. Has the participant developed functional dependence on and/or phenomenological identification with the device? Indicators may include:

- Self‑report of device feeling “transparent” or “like part of me”;
- Functional reliance on the device for daily activities;
- Expression of distress at the prospect of device removal;
- Attribution patterns that reflect embodied agency (for example, blaming oneself for device errors).

Standardised instruments such as the PIADS [63] or the QUEST 2.0 [64] may be employed to supplement qualitative assessment. *If no*: Standard trial termination or clinical protocols apply, with routine consent to explantation. *If yes*: Proceed to Step 2.

- Step 2: Assess retention safety and the proportionality of removal. Would retaining the device pose a significant and unmanageable health risk to the participant, and would removal create serious functional or psychological harm? Safety should be understood broadly. Considerations include:

- Medical risks associated with long-term implantation, including infection, electrode migration, tissue reaction, or other device-related complications;
- Device malfunction potential and availability of monitoring;
- Battery life, replacement feasibility, and the risks of further surgical intervention;
- Compatibility with future medical interventions;
- Psychological harms associated with both retention and removal, including anxiety about likely device failure or lack of technical support, as well as grief, loss of agency, identity-related distress, or depressive symptoms following removal where the device has become functionally and phenomenologically integrated.

These psychological harms should not automatically override medical safety concerns, but they should be included in the proportionality assessment and in planning psychological support.

#### If retention poses a significant and unmanageable medical risk

Explantation may be ethically required, but the decision should include comprehensive psychological support, advance preparation, and clear acknowledgement of the loss experienced by the participant.

#### If retention does not pose such a risk

Proceed to Step 3.

- Step 3: Assess participant preference. Has the participant expressed a clear, voluntary preference regarding retention or removal, informed by their post‑implantation experience? This assessment should:

- Evaluate the participant’s capacity to make this decision;
- Ensure that the preference is voluntary and not the product of coercion or undue influence;
- Document the preference in a formal re‑consent process.

#### If the participant has decision-making capacity and expresses a clear preference

This preference should be accorded substantial weight in the final determination.

#### If the participant lacks decision-making capacity

A surrogate decision-maker should be involved, guided by the participant’s known values, prior statements, and best interests.

#### If the participant retains decision-making capacity but expresses ambivalence

Ambivalence should not be treated as incapacity. It should trigger supported decision-making, additional counselling, time for reflection, and, where appropriate, consultation with family members or caregivers chosen by the participant.

The algorithm therefore treats surrogate decision-making as a response to impaired capacity, whereas ambivalence calls for enhanced support for the participant’s own decision. Once the participant’s preference, surrogate judgment, or supported decision-making process has been documented, proceed to Step 4.

- Step 4: Assess feasibility of continued support. Are there realistic plans for ongoing technical support, including:

- Battery replacement or recharging protocols;
- Software updates and cybersecurity maintenance;
- Clinical monitoring for adverse events;
- Clear designation of responsible parties (for example, research team, clinical care team, device manufacturer).

#### If no

The participant must be fully informed of the implications of retention without support, including the risk of device failure and potential need for emergency explantation, and the decision should be revisited accordingly.

#### If yes

Proceed to Step 5.

- Step 5: Reach determination. Where retention is preferred, safe, and feasible, the default should be retention, with:
- A clear plan for ongoing support and monitoring;
- Documented re‑consent acknowledging the participant’s informed choice;
- Regular reassessment at intervals appropriate to the participant’s clinical situation.
- Where retention is unsafe or infeasible, explantation should proceed, but with:

- Comprehensive psychological support before, during, and after explantation;
- Clear acknowledgement of the loss experienced by the participant;
- Consideration of alternative technologies or supports to mitigate the functional and psychological impact of removal.

**Fig. 1:**
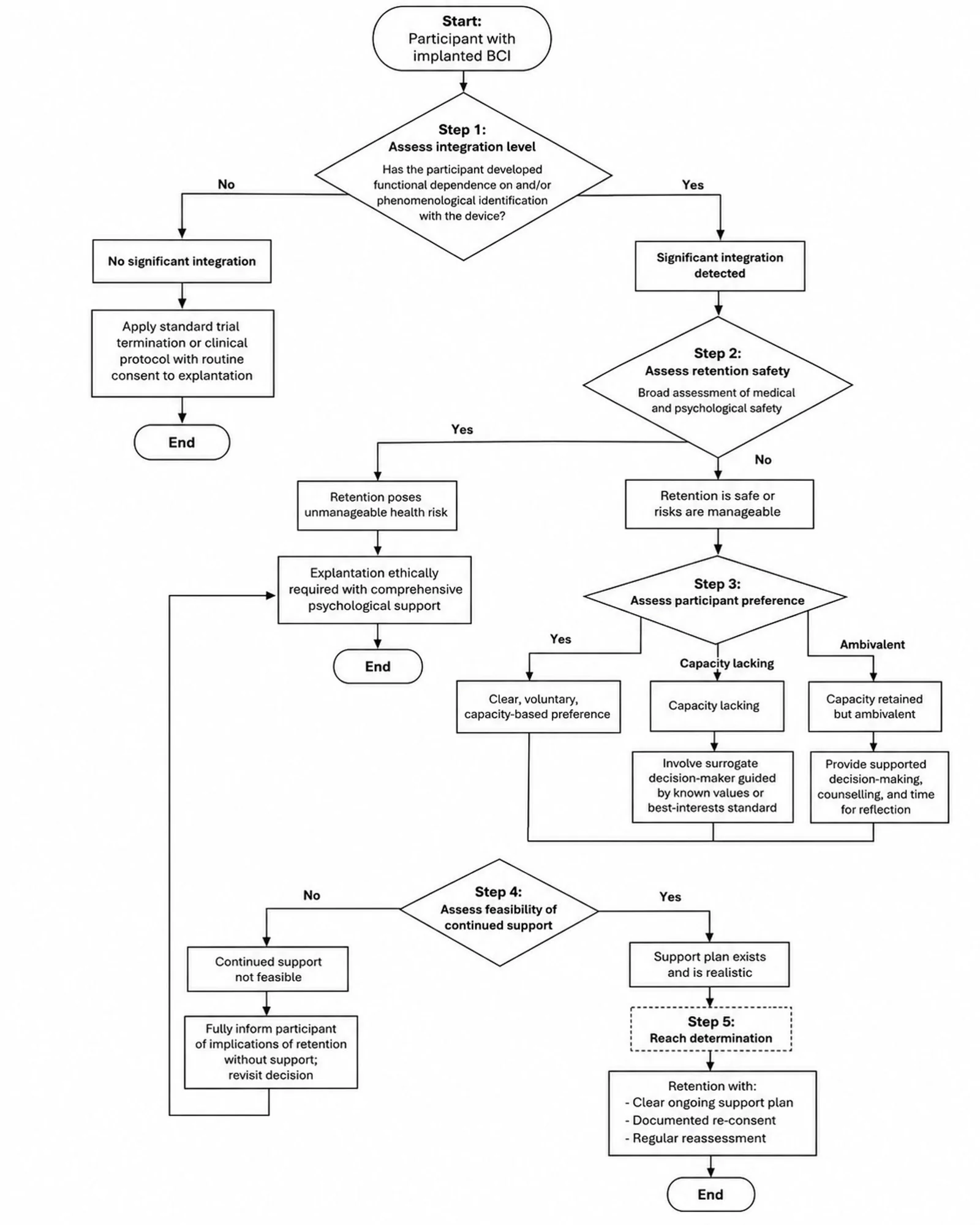
Ethical algorithm for explantation decisions in BCI research and clinical practice. The algorithm operationalizes the three proposed reforms (dynamic consent, ongoing ethical review, and decoupling withdrawal from explantation) into a structured decision pathway. Step 1 assesses phenomenological and functional integration. Step 2 evaluates safety risks of long-term retention. Step 3 ascertains the participant’s current informed preference. Step 4 confirms the feasibility of continued technical and clinical support. Step 5 reaches a determination that prioritizes the participant’s embodied experience while safeguarding against unmanageable harms

This algorithm does not eliminate the ethical complexity of explantation decisions. The very nature of transformative technologies is that they generate situations that resist algorithmic resolution. However, the algorithm provides a structured, transparent process that centres the participant’s embodied experience and evolving preferences, a marked improvement over current practice, which often defaults to removal without meaningful consideration of the participant’s transformed self. A schematic representation of this decision pathway is provided in Fig. 1.

### Feasibility considerations

A reasonable objection to the proposed reforms is that they impose an unrealistic administrative burden on ethics committees and research institutions. This concern merits serious attention but should not be overstated. First, the tracking review mechanism is intended only for invasive BCI trials involving long‑term implantation; non‑invasive studies and short‑term trials would continue under standard review procedures. Second, the frequency of reviews can be calibrated to the trial’s phase—for example, a review at reliable device control, another at six months, and annually thereafter, amounting to three to four additional reviews over a multi‑year implantation period. Third, the emergence of specialized neuroethics committees and the increasing availability of BCI ethics expertise in China suggest growing institutional capacity. The 2025 Implementation Opinions jointly issued by seven government departments explicitly call for “continuously promoting ethical research” [55], signalling policy‑level recognition of the need for enhanced oversight. Fourth, decoupling withdrawal from explantation does not itself create new review burdens; it merely clarifies a distinction poorly operationalized under current frameworks. Finally, the cost of not implementing these reforms must also be weighed: participant abandonment, psychological harm from non‑voluntary explantation, and erosion of public trust in BCI research. On balance, the incremental burden of targeted, risk‑stratified ongoing review is justified by the significant ethical harms it aims to prevent.

### Limitations and future directions

This analysis has several limitations that should be acknowledged. First, the empirical evidence base on the long-term psychological and identity effects of BCI explantation remains limited. While qualitative studies provide rich phenomenological data, larger-scale longitudinal research is needed to systematically characterise the prevalence and nature of embodied integration and its implications for consent. Relatedly, the argument should not be read as applying equally to all BCI technologies. It is most relevant to long-term invasive BCIs that generate functional dependence, phenomenological incorporation, or identity-related distress at the prospect of removal. Short-term experimental use, non-invasive BCIs, or implanted devices that remain clearly external to the user’s self-experience may not raise the same diachronic consent problem. Future empirical work should therefore examine which technical and experiential features make device integration ethically significant. Second, the focus on China’s regulatory framework, while timely given recent policy developments, may limit generalisability to other jurisdictions with different legal and ethical infrastructures. However, the core normative problem, namely the diachronic consent problem, is not jurisdiction-specific, and the proposed reforms can be adapted to diverse regulatory contexts. Third, the paper does not address in depth how the diachronic consent problem may vary across therapeutic, enhancement, research, and commercial BCI contexts. These contexts may involve different expectations of benefit, different institutional responsibilities, and different forms of participant or consumer vulnerability. Fourth, the analysis focuses primarily on the participant’s perspective; further research should explore the perspectives of caregivers, family members, and clinicians, who are also affected by BCI implantation and explantation decisions. Finally, the proposed ethical algorithm requires validation through implementation and evaluation in real-world clinical and research settings.

## Conclusions

The clinical translation of invasive BCI technologies in China marks an important moment in neurotechnology governance. Recent developments surrounding the NEO system and the establishment of ethical, regulatory, reimbursement, and clinical frameworks show that BCI is moving from experimental research toward more routine clinical and possibly commercial use. This transition makes one ethical question increasingly urgent: how should consent be understood when a participant initially agrees to the later removal of a device that may, through long-term use, become functionally and phenomenologically integrated into her agency?

This paper has argued that the diachronic consent problem cannot be adequately addressed by a single-point consent model. The problem does not arise simply because BCI use may be transformative. Many medical and life experiences are transformative in relevant respects. The distinctive concern arises where long-term invasive BCI use combines ex ante experiential ignorance with direct neural coupling, functional dependence, embodied agency, and phenomenological incorporation. In such cases, initial consent to explantation should remain ethically important, but it should not be treated as conclusively authorising later removal without renewed assessment of the participant’s post-implantation experience and current preference.

Dynamic consent, ongoing ethical review, and the decoupling of withdrawal from research from consent to explantation provide a more responsive framework. These reforms would not require that every implanted device be retained, nor would they deny the importance of medical safety, research integrity, or institutional feasibility. Rather, they would ensure that explantation decisions take seriously the participant’s evolving embodied experience, the psychological and functional consequences of removal, and the responsibilities created by long-term neural device research. As BCI technologies continue to develop, ethical governance must not remain fixed at the moment of initial signature; it must be capable of following the participant through the changing relationship between body, agency, and device.

## Data Availability

All data generated or analysed during this study are included in this published article. No new empirical datasets were generated or analysed during the current study. The analysis is based on publicly available regulatory documents, ethical guidelines, and previously published scholarly literature, all of which are cited in the manuscript. Accordingly, no additional raw data are available for sharing.

## Abbreviations

ALS: Amyotrophic lateral sclerosis
BCI: Brain‑computer interface
CIOMS: Council for International Organizations of Medical Sciences
EEG: Electroencephalography
GDPR: General Data Protection Regulation
NEO: Neural Epidural Observation (system)
PIADS: Psychosocial Impact of Assistive Devices Scale
PIAAS: Personality, identity, autonomy, authenticity, agency, and self
QUEST: Quebec User Evaluation of Satisfaction with assistive Technology
UNESCO: United Nations Educational, Scientific and Cultural Organization
